# Electrical impedance tomography in pediatric patients with COVID-19, the first reports

**DOI:** 10.1186/s12890-021-01716-y

**Published:** 2021-11-08

**Authors:** Milena S. Nascimento, Glasiele C. Alcala, Ana I. A. Guzman, Leticia C. Corrêa, Diana M. Baggio, Felipe S. Rossi, Linus P. Fascina, Marcelo B. P. Amato, Cristiane do Prado

**Affiliations:** 1 Department of Pediatrics, Hospital Israelita Albert Einstein, Albert Einstein Avenue, 627-701, São Paulo, SP 05651-901 Brazil; 2 Developer Division, Timpel SA, São Paulo, Brazil; 3 Divisão de Pneumologia, Cardiopulmonary Department, Heart Institute (INCOR), São Paulo, SP Brazil

**Keywords:** Electrical impedance tomography, Computed tomography, COVID-19, Pediatric intensive care

## Abstract

**Introduction:**

Electrical impedance tomography (EIT) is a noninvasive, radiation-free, bedside tool to monitor ventilation distribution in real time.

**Objective:**

To evaluate, in pediatric COVID-19 patients, the ventilation distribution using EIT and compare it to thoracic computed tomography (TCT) or chest radiograph results obtained in these patients.

**Methods:**

This was a prospective, observational clinical study including pediatric patients admitted to the intensive care unit of a private hospital. The patients monitored with EIT tested positive for COVID-19 and were submitted to the previously mentioned radiation exams. EIT monitoring lasted 15 min and no sedation was used.

**Results:**

Six patients were included in this study. The main differences observed in the EIT were in the right-left distribution and were compatible with the morphological changes found in the TCT or radiograph images due to COVID-19 infection.

**Conclusion:**

We conclude that EIT is ready to investigate the ventilatory profile present at different lung diseases, including COVID-19, and might postpone or mitigate the need of repeated ionizing radiation exams in the pediatric population, although larger pediatric cohorts comparing to standard radiological imaging are needed.

## Introduction

In December 2019, a set of pneumonia cases, which were later proven to be caused by a new coronavirus (named “COVID-19”), appeared in the city of Wuhan, Hubei Province, China and was declared a pandemic by the World Health Organization in March 2020 [1, 2].

In contrast to adult data, children present asymptomatic infection in 13% of virologically confirmed cases, and only 0.6% progress to acute respiratory distress syndrome (ARDS) or multiple organ dysfunction [3]. A different inflammatory response profile among these younger age groups seems to determine this less severe disease presentation [4].

Although widely used in the adult population, thoracic computed tomography (TCT) exams to evaluate noncritical patients are less frequently performed in the pediatric population, due to ionizing radiation concerns and the need of patient collaboration [5]. However, during the COVID-19 pandemic and following the guidelines adopted in adult patients, pediatric TCT exams were more frequently achieved [6]. Recent TCT scan findings of five pediatric patients were milder but in accordance with the abnormalities depicted in adult COVID-19 patients [7].

Electrical impedance tomography (EIT) allows radiation-free, noninvasive portable bedside respiratory monitoring [8]. Regional pulmonary ventilation, cycle by cycle and in real time, is demonstrated with EIT application and characterizes the changes in the ventilation distribution [8–11]. We hypothesized that EIT to assess pulmonary ventilation in pediatric patients with COVID-19 might expand the knowledge on this disease and guide respiratory interventions in advance.

## Objectives

To evaluate the ventilation distribution using EIT and relate it to TCT or to chest radiograph findings in COVID-19 pediatric patients.

## Patients and methods

### Study type, location and patients

This was a prospective, observational clinical study including pediatric patients admitted to the intensive care unit of a private hospital in Sao Paulo, Brazil, from April 1st to December 31st 2020. The patients underwent TCT scans or chest radiograph exams and tested positive for COVID-19 (RT-PCR for SARS CoV-2). The study was approved by the Hospital Research Ethics Committee registered with the number 30842420.3.0000.0071, and an informed consent form was signed by the parents.

### Availability of data and materials

All data generated or analyzed during this study are included in this published article, except original image files as they contain patient identification data. The data that support the findings of this study are available on request from the corresponding author [MSN]. The data are not publicly available due to “them containing information that could compromise research participant privacy/consent”.

### Protocol

The EIT acquisition belt was installed at the mammillary line (24 or 32 electrodes, according to the infant's chest circumference. ENLIGHT 1800, Timpel, São Paulo, Brazil), with patients in the supine position. The EIT monitoring lasted 15 min and no sedation was necessary.

The record of the electrical impedance variation over time is called a plethysmogram, and this provides two pieces of information: the impedance variation, called delta Z ($\Delta$Z), which has an excellent correlation with the volume variation evaluated by computed tomography, and the minimum impedance (EELZ), which is the baseline of the plethysmogram and corresponds to the functional residual capacity (FRC). EIT allows regional ventilation evaluation (analyzing pixel by pixel variations, in a 32 × 32 mesh). The images were divided into four regions of interest (ROIs), anterior, posterior, right and left, and the ROIs with greater variations in $\Delta$Z correspond to the most ventilated segments, while lower levels of $\Delta$Z amplitude correspond to lower regional ventilation [11].

## Results

During the study period, seven patients were admitted to the pediatric intensive care unit (PICU) after testing positive for COVID-19, and six were included in this study. One patient was excluded due to an error in retrieving the EIT file. Demographic characteristics and concise radiation exams findings are described in Table 1.

**Table 1:** Demographic characteristics and radiation exams findings of the patients

Patients	Age (months)	Weight (kg)	PIM 2 (%)	Clinical Signs	Ventilatory support	TCT or chest radiograph
1	17	12	1.1	Asymptomatic	Room air	Minimal diffuse ground-glass opacities and slight right pleural effusion
2	7	7.5	1.6	Asymptomatic	Room air	Ground-glass opacities in the left lung, mainly in the lingula
3	122	57	1.1	Coryza/cough	Oxygen therapy (1 L/O_2_)	Elevated left diaphragm
4	84	15	3.5	Respiratory distress	Noninvasive ventilation	Atelectasis inferior left lobe
5	3	7.8	0.4	Asymptomatic	Room air	No abnormalities
6	132	33	1.8	Abdominal symptoms	Room air	No abnormalities

PIM 2, Pediatric Index of Mortality; TCT, thoracic computed tomography; TCT, patients 1 and 2, chest radiograph: patients 3, 4, 5 and 6

As presented in Table1, most patients were asymptomatic or had a mild disease presentation. No patients presented clinical complication seen after the EIT images were collected. The length of stay ranged from 2 to 4 days, except for patient 4 who remained in the hospital for 16 days. Patient 4 presented respiratory insufficiency and was treated with non-invasive ventilation for eleven days, and discharged home after 16 days.

The EIT detected the right-left distribution asymmetries in patients 2, 3 and 4. Patient 2 TCT presented a less aerated left lung in comparison to the right side. Patients 3 and 4 CR demonstrated an altered pattern, both on the left side (elevated diaphragm and inferior atelectasis, respectively) (Table 1). The EIT findings in the remaining patients, 1, 5 and 6, were unremarkable.

The TCT of patient 1 presented minimal diffuse ground-glass opacities evenly distributed over the lungs and a slight right pleural effusion. Patients 5 and 6 had normal considered chest radiograph.

**Fig. 1:**
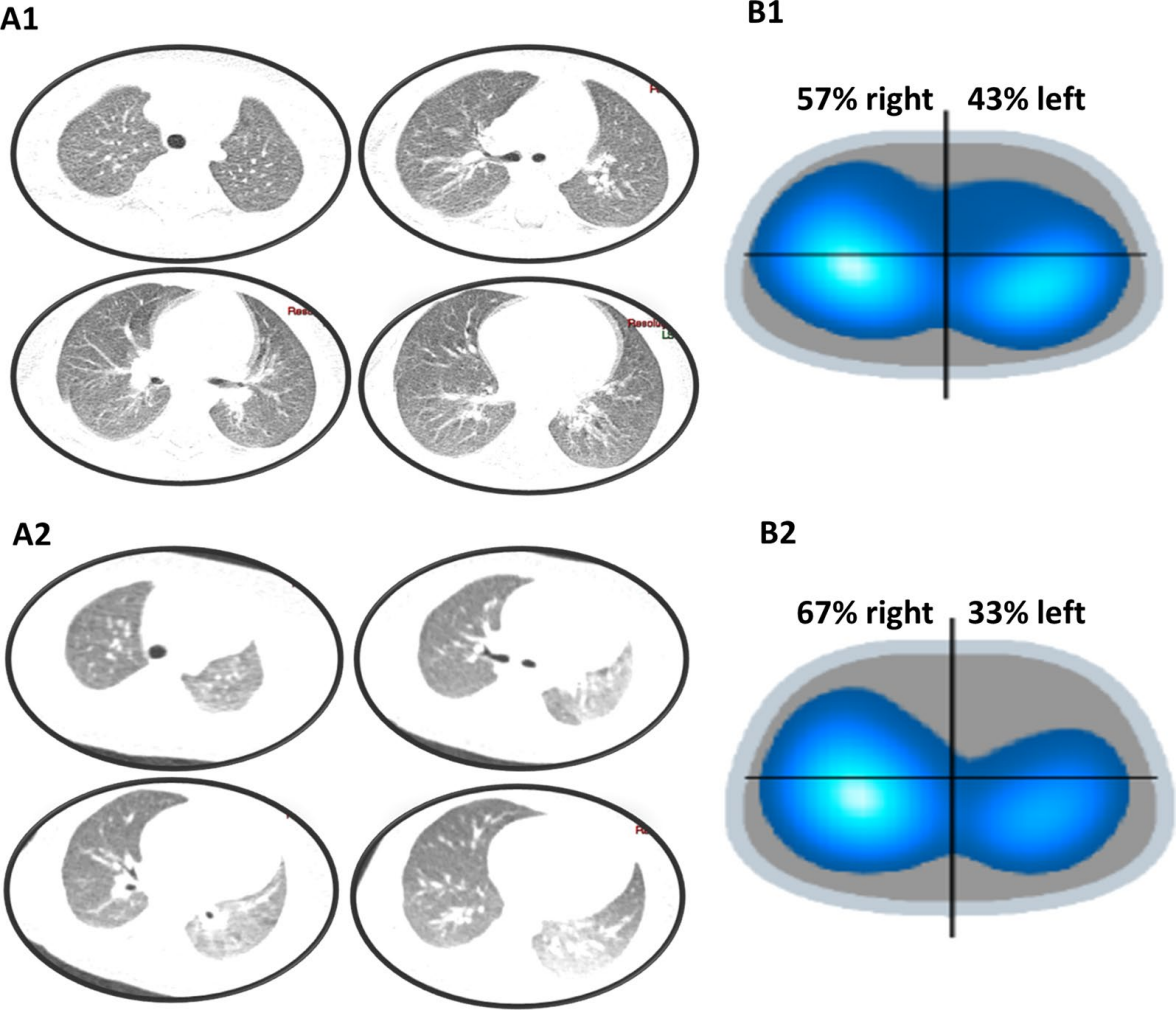
The top images correspond to patient 1, a 17 months-old girl. The lower images correspond to patient 2, a seven months-old boy. **A1** and **A2** shows thoracic computed tomography (TCT) images. The EIT images **B1** and **B2** are divided into four ROIs based on the electrodes placed on the monitoring belt. The color scale of the EIT corresponds to the tidal volume variation present at the different ROIs; dark blue corresponds to a lower ventilation variation, and white corresponds to high tidal volumes. TCT of patient 1 had minimal ground-glass opacities close to the right pleural effusion, probably related to a restrictive atelectatic component. Absence of other focal pulmonary opacities suggestive of a parenchymal infectious process. The EIT demonstrated the usual ventilatory distribution profile. TCT of patient 2 had ground-glass pulmonary opacities in the left lung, mainly in the lingula and lower lobe, which may represent a viral inflammatory/infectious process in the clinical context presented, although there is a possibly contributing hypoexpansion component for the frosted glass appearance. There are no alveolar consolidations. There was also a slight accumulation of secretion inside some bronchi in the left lung base. The EIT of this patient, who showed decreased ventilation in the left lung, was consistent with the TCT findings

The TCT are represented in Fig. 1, including EIT and TCT findings from patients 1 and 2.

**Fig. 2:**
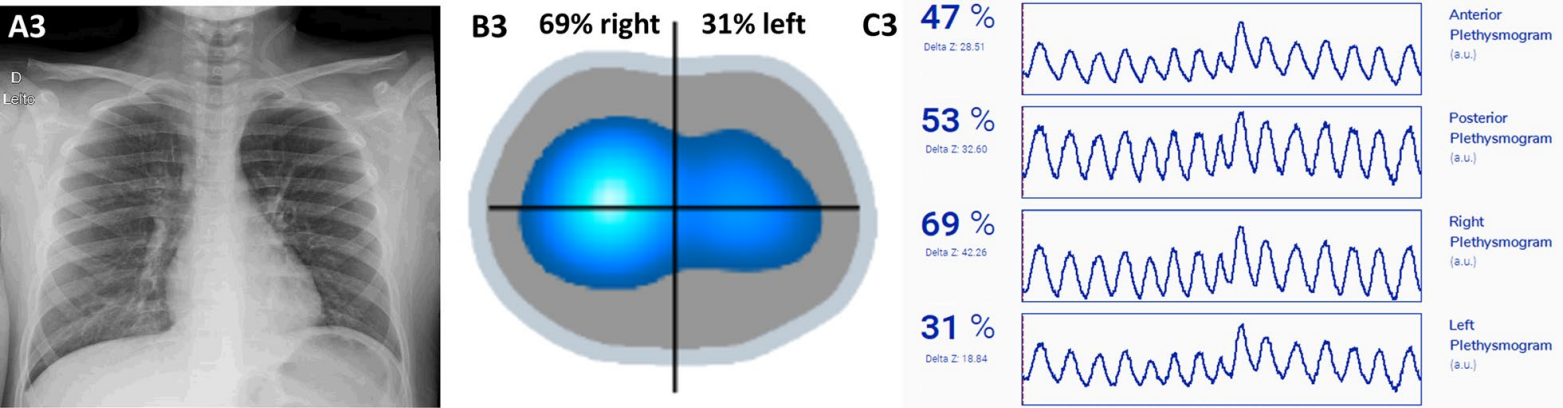
The figure shows chest radiograph images (**A3**), EIT images (**B3**) and plethysmogram (**C3**) of patient 3, a 10 years-old boy. The radiograph image shows elevation of the left diaphragm. EIT showed less variation in ventilation in the left region, compatible with the radiological image. The color scale of the EIT corresponds to the tidal volume variation present at the different ROIs; dark blue corresponds to a lower ventilation variation, and white corresponds to high tidal volumes. The plethysmogram (**C3**) shows the distribution of ventilation in the 4 ROis (upper, lower, right and left), and the amplitude of the curve indicates the variation in ventilation (delta Z), which is greater on the right lung (69%) than on the left lung (31%)

**Fig. 3:**
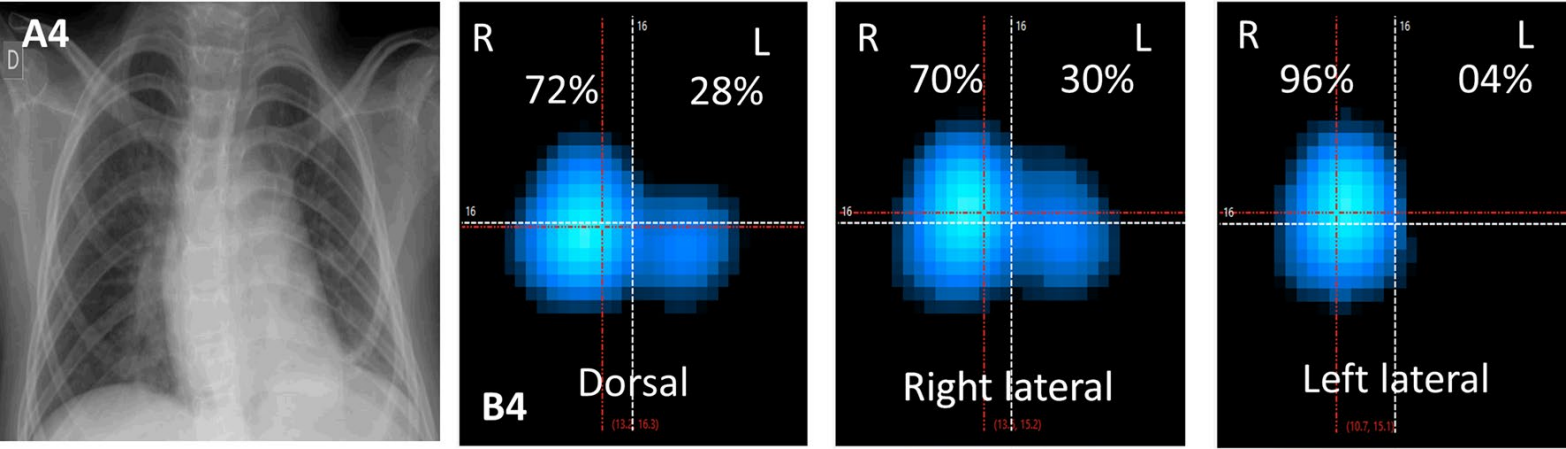
The figure shows chest radiograph images and EIT images of patient 4, a four years-old girl, in 3 different decubitus: dorsal, right lateral and left lateral. The patient was using NIV, through a face mask, with an inspiratory pressure of 20 cmH_2_O and a positive end-expiratory pressure of 8 cmH_2_O. The radiograph image **A4** show clamping of costal arches and elevation of the left diaphragmatic dome suggestive of atelectasis. The EIT, other three panels, shows the distribution of ventilation according to the patient's position. The color scale of the EIT corresponds to the tidal volume variation present at the different ROIs; dark blue corresponds to a lower ventilation variation, and white corresponds to high tidal volumes. In the supine position (**B4**), a decrease in ventilation on the left lung is observed, compatible with the radiological image. When the right decubitus position was adopted, a ventilation increase in the dependent right lung was observed, consistent with the physiological response. When the left lung was in the dependent position, left lateral decubitus, almost no ventilation in this lung was detected (4%). Most likely, the action of gravity leads to the dynamic closure of small airways, limiting the flow, and preventing ventilation

The Figs. 2 and 3 presents the EIT and chest radiograph images from patients 3 and 4, respectively. Both EIT depicts ventilation asymmetry, with less ventilation on the left side.

**Fig. 4:**
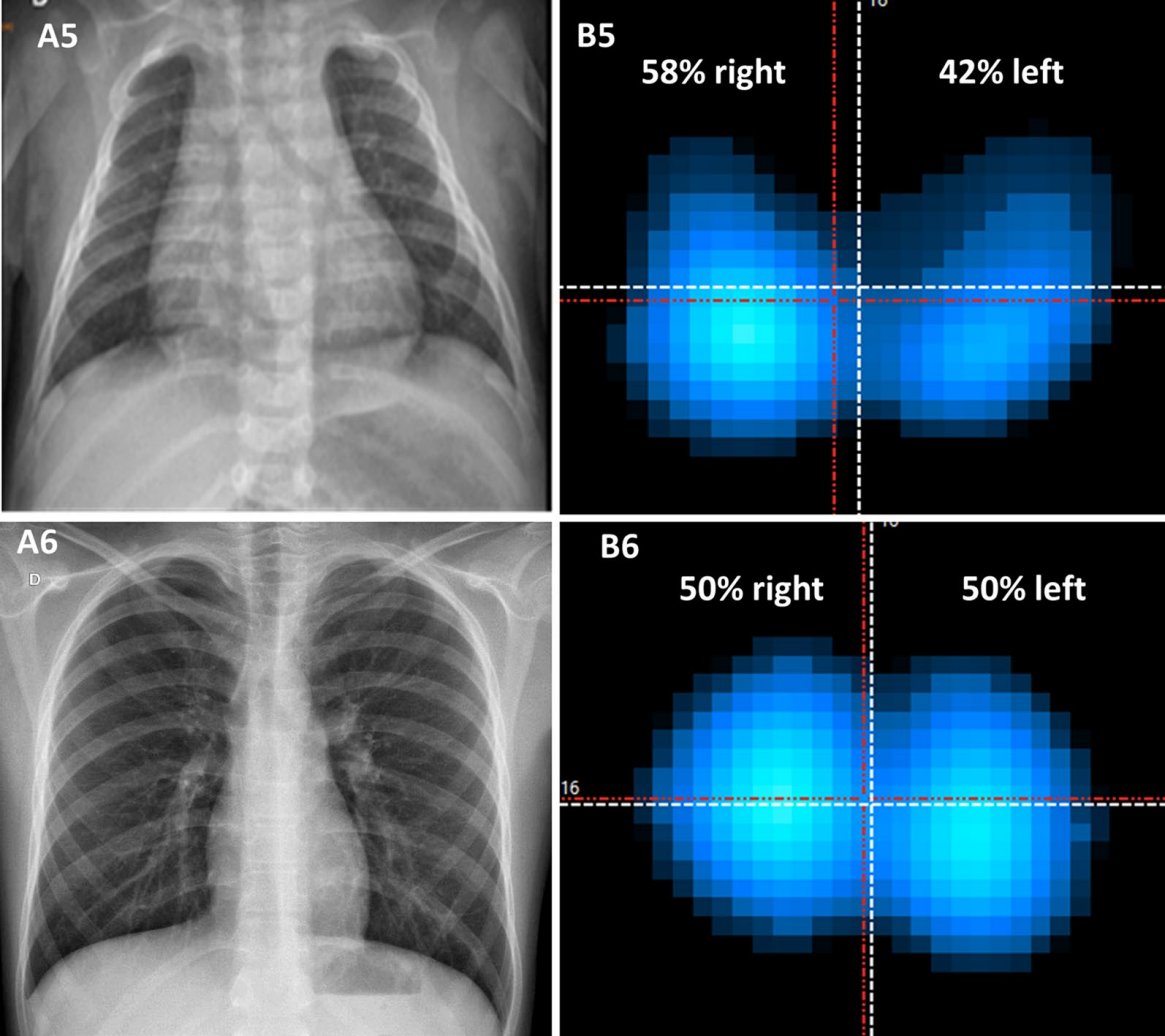
Chest radiograph images (**A**) and EIT images (**B**) of patient 5 and patient 6. The patient 5 was a three months-old girl and the patient 6 an 11 years-old boy. The radiograph images of both patients were normal, and EIT also showed no changes in ventilation distribution. The color scale of the EIT corresponds to the tidal volume variation present at the different ROIs: dark blue corresponds to a lower ventilation variation, and white corresponds to high tidal volumes

Figure 4 presents usually expected ventilation distribution EIT findings, and their correspondent radiograph, with normal appearance.

## Discussion

Our study provides the first results relating EIT ventilation profiles to TCT or chest radiograph findings in COVID-19 pediatric patients.

Recently published data on EIT use in the pediatric population states that this technology “seems to be predestined for the clinical use in neonates, infants and children”, since it is a radiation free imaging tool that does not need patient collaboration. Moreover, EIT can be used for prolonged periods, at the bedside, in the PICU and NICU environments. This study points the EIT ability to explore air distribution, to determine the center of ventilation, to analyze the inhomogeneity index of air distribution and estimate the tidal volume and FRC, aspects that are considered highly significant to the researchers using this technology, some of them presented at our data on COVID-19 children [12].

Previous validation data confirm that EIT is a highly reproducible method in which impedance changes accurately reflect regional ventilation variations [9, 11]. The EIT validation study compared impedance evaluation results, initially considered as a lung functional image, to TCT images, considered a hallmark of anatomic air distribution within the lungs, and with a very consistent result [9].

The ventilation distribution ratio present in the asymptomatic patients in our study was in accordance to previously published data in spontaneously breathing healthy children, where pulmonary ventilation distribution is predominantly directed to the posterior (65–70%) and right (52–55%) lung regions [13, 14]. Nevertheless, different data observed that the ventilation pattern in healthy children is quite variable when compared to a more well-established pattern reported in adults [13].

In our presented cases, the abnormal ventilatory profiles found, particularly in the right-left distribution, agreed with the morphological changes found in the TCT or the chest radiograph images due to COVID-19 infection. Li W et al. described the findings of five children positive for COVID-19, four of whom were asymptomatic, and one presented only mild respiratory symptoms. Two patients had no abnormalities detected through TCT, while the other three presented a very modest form of ground-glass opacification [7]. Of the six patients in our study, two underwent chest TCT and had a ground-glass pattern, and one of them had pleural effusion. The EIT pattern differed between them, since patient 2 presented a more uneven distribution of the disease which affected more the left lower lung (Fig. 1).

Although the performance of the TCT was also recommended for the pediatric population as one of the clinical criteria for the diagnosis of COVID-19 early in the pandemic, some points were reconsidered for the non-routine performance of the exam. The images in the pediatric population are more nonspecific than in adults to be used as a diagnostic criterion, and in many cases with confirmed RT-PCR for the virus, TCT images are normal [15, 16]. In addition, radiation exposure should be considered for the indication and need for TCT [17]. Additionally, recent concerns were raised in the pediatric population exposed to ionizing radiation, since there is evidence of increased cancer development in this population [18, 19]. According to the Guideline for Medical Imaging in Auxiliary Diagnosis of COVID-19, the accompanying images can be made by radiograph exams [15].

EIT is radiation-free, portable, and easily performed at the bedside, and can be useful in the management and evaluation of pediatric patients with respiratory diseases, including COVID-19. In addition, the EIT data on ventilation distribution and EELZ, a surrogate of functional residual capacity, provides new information on patient monitoring and can assist titration of the respiratory support. In one of our presented cases, patient 4 needed noninvasive support due to respiratory failure and was evaluated in three positions. The EIT information demonstrated that the left lung ventilation was strongly compromised under the action of gravity, and left lateral decubitus was avoided, possibly helping in the patient outcome.

Recently EIT published data depicted self-inflicted lung injury mechanism, named “*Pendelluft*”, in a P-ARDS infant and in a preterm infant treated with minimally invasive surfactant therapy [20, 21]. In those low compliance respiratory illness, EIT can monitor changes in air distribution and detect the lung recruitment at the bedside, as was already reported in adults both in the ICU and during anesthesia [22, 23]. Although large series are not yet available in the pediatric population, we believe that EIT help tailoring ventilatory support in many situations based on the different reports already published [24–26].

One of the limitations of our study was the small number of children included due to the reduced number of children hospitalized secondary to COVID-19, since a milder disease in children is the usual presentation [27, 28]. In our service, there were only seven hospitalizations in the PICU during the study period, and this was shown to be strongly determined by the social isolation measures implemented in Sao Paulo to contain new COVID-19 cases during the pandemic, including closing schools and daycare centers, which drastically interfered with the seasonality of respiratory diseases in the pediatric population [29].

## Conclusion

The presented data demonstrate the pulmonary ventilation distribution in six pediatric patients with COVID-19 detected by EIT. The ventilatory profiles observed during the EIT monitoring were compatible with the clinical presentation of the patients, and the morphological abnormalities present in the TCT or chest radiograph images were EIT characterized. We conclude that EIT is ready to investigate, at bedside and in real-time, the ventilatory profile present at different lung diseases, including COVID-19, and might postpone or mitigate the need of repeated ionizing radiation exams in the pediatric population, although larger pediatric cohorts comparing to standard radiological imaging are needed.

## Data Availability

Not applicable.

## Abbreviations

EIT: Electrical impedance tomography
TCT: Thoracic computed tomography
ARDS: Acute respiratory distress syndrome
FRC: Functional residual capacity
ROIs: Regions of interest
PICU: Pediatric intensive care unit
PIM 2: Pediatric index of mortality

## Declarations

### Category of study

This manuscript is identified as special article.

## References

[1] Zhu N, Zhang D, Wang W, Li X, Yang B, Song J (2020). A novel coronavirus from patients with pneumonia in China, 2019. N Engl J Med.

[2] World Health Organization. Coronavirus disease (COVID-2019) situation reports. Available from:https://www.who.int/emergencies/diseases/novel-coronavirus-2019/situation-reports. Accessed 18 Mar 2020.

[3] Dong Y, Mo X, Hu Y (2020). Epidemiology of COVID-19 among children in China. Pediatrics.

[4] Schouten LR, van Kaam AH, Kohse F (2019). Age-dependent differences in pulmonary host responses in ARDS: a prospective observational cohort study. Ann Intensive Care.

[5] Pan Y, Guan H, Zhou S, Wang Y, Li Q, Zhu T, Hu Q, Xia L (2020). Initial CT findings and temporal changes in patients with the novel coronavirus pneumonia (2019-nCoV): a study of 63 patients in Wuhan, China. Eur Radiol.

[6] Shen K, Yang Y, Wang T, Zhao D, Jiang Y, Jin R, Zheng Y (2020). Diagnosis, treatment, and prevention of 2019 novel coronavirus infection in children: experts’ consensus statement. World J Pediatr.

[7] Li W, Cui H, Li K, Fang Y, Li S (2020). Chest computed tomography in children with COVID-19 respiratory infection. Pediatr Radiol.

[8] Frerichs I (2000). Electrical impedance tomography (EIT) in applications related to lung and ventilation: a review of experimental and clinical activities. Physiol Meas.

[9] Victorino JA, Borges JB, Okamoto VN, Matos GFJ, Tucci MR, Caramez MPR, Tanaka H, Sipmann FS, Santos DCB, Barbas CSV, Carvalho CRR, Amato MBP (2004). Imbalances in regional lung ventilation. A validation study on electrical impedance tomography. Am J Respir Crit Care Med.

[10] Frerichs I, Schiffmann H, Hahn G, Hellige G (2001). Noninvasive radiation-free monitoring of regional lung ventilation in critically ill infants. Intensive Care Med.

[11] Frerichs I, Amato MB, van Kaam AH, Tingay DG, Zhao Z, Grychtol B, Bodenstein M, Gagnon H (2016). Chest electrical impedance tomography examination, data analysis, terminology, clinical use and recommendations: consensus statement of the TRanslational EIT developmeNt stuDy group. Thorax.

[12] Frerichs I, Becher T (2019). Chest electrical impedance tomography measures in neonatology and paediatrics-a survey on clinical usefulness. Physiol Meas.

[13] Lupton-Smith AR, Argent AC, Rimensberger PC, Morrow BM (2014). Challenging a paradigm: positional changes in ventilation distribution are highly variable in healthy infants and children. Pediatr Pulmonol.

[14] Pham TM, Yuill M, Dakin C, Schibler A (2011). Regional ventilation distribution in the first 6 months of life. Eur Respir J.

[15] Li HJ, Liu SY, Xu HB (2019). Cheng JL (2020) Guideline for medical imaging in auxiliary diagnosis of coronavirus disease. Chin J Med Imaging Technol.

[16] Xia W, Shao J, Guo Y, Peng X, Li Z, Hu D (2020). Clinical and CT features in pediatric patients with COVID-19 infection: different points from adults. Pediatr Pulmonol.

[17] Duan Y, Zhu Y, Tang L (2020). CT features of novel coronavirus pneumonia (COVID-19) in children. Eur Radiol.

[18] Smith-Bindman R, Miglioretti DL, Johnson E (2012). Use of diagnostic imaging studies and associated radiation exposure for patients enrolled in large integrated health care systems, 1996–2010. JAMA.

[19] Mathews JD, Forsythe AV, Brady Z (2013). Cancer risk in 680,000 people exposed to computed tomography scans in childhood or adolescence: data linkage study of 11 million Australians. BMJ.

[20] Rossi FS, Costa ELV, Iope DDM, Pacce PHD, Cestaro C, Braz LZ (2019). Pendelluft detection using electrical impedance tomography in na infant: keep those images in mind. Am J Respir Crit Care Med.

[21] Gonçalves-Ferri WA, Rossi FS, Costa ELV, Correa L, Iope D, Dalla Pacce P (2020). Lung recruitment and pendelluft resolution after less invasive surfactant administration in a preterm infant. Am J Respir Crit Care Med.

[22] Costa EL, Borges JB, Melo A, Suarez-Sipmann F, Toufen C, Bohm SH, Amato MB (2009). Bedside estimation of recruitable alveolar collapse and hyperdistension by electrical impedance tomography. Intensive Care Med.

[23] Simon P, Girrbach F, Petroff D, Schliewe N, Hempel G, Lange M, Bluth T (2021). PROBESE investigators of the protective ventilation network* and the clinical trial network of the European Society of Anesthesiology. Individualized versus fixed positive end-expiratory pressure for intraoperative mechanical venti-lation in obese patients: a secondary analysis. Anesthesiology.

[24] Miedema M, Frerichs I, de Jongh FH, van Veenendaal MB, van Kaam AH (2011). Pneumothorax in a preterm infant monitored by electrical impedance tomography: a case report. Neonatology.

[25] Hough JL, Johnston L, Brauer SG, Woodgate PG, Pham TM, Schibler A (2012). Effect of body position on ventilation distribution in preterm infants on continuous positive airway pressure. Pediatr Crit Care Med.

[26] Bhatia R, Carlisle HR, Armstrong RK, Kamlin COF, Davis PG, Tingay DG. Extubation generates lung volume inhomogeneity in preterm infants. Arch Dis Child Fetal Neonatal Ed. 2021:321788. 10.1136/archdischild-2021-321788 34162692

[27] Chen ZM, Fu JF, Shu Q, Chen YH, Hua CZ, Li FB (2020). Diagnosis and treatment recommendations for pediatric respiratory infection caused by the 2019 novel coronavirus. World J Pediatr.

[28] Alsohime F, Temsah MH, Al-Nemri AM, Somily AM, Al-Subaie S (2020). COVID-19 infection prevalence in pediatric population: etiology, clinical presentation, and outcome. J Infect Public Health.

[29] Nascimento MS, Baggio DM, Fascina LP, do Prado C (2020). Impact of social isolation due to COVID-19 on the seasonality of pediatric respiratory diseases. PLoS ONE.

